# Running and Physical Activity in an Air-Polluted Environment: The Biomechanical and Musculoskeletal Protocol for a Prospective Cohort Study 4HAIE (Healthy Aging in Industrial Environment—Program 4)

**DOI:** 10.3390/ijerph17239142

**Published:** 2020-12-07

**Authors:** Daniel Jandacka, Jaroslav Uchytil, David Zahradnik, Roman Farana, Dominik Vilimek, Jiri Skypala, Jan Urbaczka, Jan Plesek, Adam Motyka, Denisa Blaschova, Gabriela Beinhauerova, Marketa Rygelova, Pavel Brtva, Klara Balazova, Veronika Horka, Jan Malus, Julia Freedman Silvernail, Gareth Irwin, Miika T. Nieminen, Victor Casula, Vladimir Juras, Milos Golian, Steriani Elavsky, Lenka Knapova, Radim Sram, Joseph Hamill

**Affiliations:** 1 Human Motion Diagnostic Center, Department of Human Movement Studies, University of Ostrava, 70200 Ostrava, Czech Republic; Jaroslav.uchytil@osu.cz (J.U.); David.zahradnik@osu.cz (D.Z.); roman.farana@osu.cz (R.F.); dominik.vilimek@osu.cz (D.V.); jiri.skypala@osu.cz (J.S.); jan.urbaczka@osu.cz (J.U.); jan.plesek@osu.cz (J.P.); adam.motyka@osu.cz (A.M.); Denisa.blaschova@osu.cz (D.B.); gabriela.beinhauerova@osu.cz (G.B.); marketa.rygelova@osu.cz (M.R.); pavel.brtva@osu.cz (P.B.); klara.balazova@osu.cz (K.B.); veronika.horka@osu.cz (V.H.); jan.malus@osu.cz (J.M.); GIrwin@cardiffmet.ac.uk (G.I.); milos.golian@osu.cz (M.G.); steriani.elavsky@osu.cz (S.E.); lenka.knapova@osu.cz (L.K.); jhamill@kin.umass.edu (J.H.); 2 Department of Kinesiology and Nutrition Sciences, University of Nevada Las Vegas, Las Vegas, NV 89154, USA; jfs@unlv.edu; 3 Cardiff School of Sport and Health Sciences, Cardiff Metropolitan University, Cardiff CF5 2YB, UK; 4 Research Unit of Medical Imaging, Physics and Technology, University of Oulu, FI-90014 Oulu, Finland; miika.nieminen@oulu.fi (M.T.N.); Victor.Casula@oulu.fi (V.C.); 5 High Field MR Centre, Department of Biomedical Imaging and Image-Guided Therapy, Medical University of Vienna, 1090 Vienna, Austria; vladimir.juras@meduniwien.ac.at; 6 Institute of Experimental Medicine AS CR, 142 20 Prague, Czech Republic; radim.sram@iem.cas.cz; 7 Department of Kinesiology, University of Massachusetts, Amherst, MA 01003, USA

**Keywords:** MRI, running, walking cutting, environment, knee, ankle, ACL, cartilage, achilles tendon

## Abstract

Far too little attention has been paid to health effects of air pollution and physical (in)activity on musculoskeletal health. The purpose of the Healthy aging in industrial environment study (4HAIE) is to investigate the potential impact of physical activity in highly polluted air on musculoskeletal health. A total of 1500 active runners and inactive controls aged 18–65 will be recruited. The sample will be recruited using quota sampling based on location (the most air-polluted region in EU and a control region), age, sex, and activity status. Participants will complete online questionnaires and undergo a two-day baseline laboratory assessment, including biomechanical, physiological, psychological testing, and magnetic resonance imaging. Throughout one-year, physical activity data will be collected through Fitbit monitors, along with data regarding the incidence of injuries, air pollution, psychological factors, and behavior collected through a custom developed mobile application. Herein, we introduce a biomechanical and musculoskeletal protocol to investigate musculoskeletal and neuro-mechanical health in this 4HAIE cohort, including a design for controlling for physiological and psychological injury factors. In the current ongoing project, we hypothesize that there will be interactions of environmental, biomechanical, physiological, and psychosocial variables and that these interactions will cause musculoskeletal diseases/protection.

## 1. Introduction

The health effects of poor air quality are unlikely to be limited to cardiovascular and respiratory problems. Far too little attention has been paid to the health effects of air pollution on the musculoskeletal system. Musculoskeletal problems represent a global threat to healthy aging and can lead to the development of musculoskeletal diseases [1]. Reduced physical function often leads to mental health decline, increased risk of developing other chronic diseases, and increased all-cause mortality [1,2]. Recently, the English Longitudinal Study of Aging described a possible link between air pollution exposure and cartilage damage in the English population [3]. Therefore, the health effects of air pollution on the musculoskeletal system need further investigation. The English Longitudinal Study of Aging did not directly measure tissue quality, air pollution associated with participants’ physical activity, or the biomechanical load of musculoskeletal system [3]. To understand the effect of air pollution on the musculoskeletal system, it may be helpful to examine regularly physically active and inactive individuals permanently living in an air-polluted versus unaffected (control) regions.

Air pollution and health: Active transportation provides a substantial health benefit because of increased physical activity (PA) [4]. However, active travel may increase the intake of air pollution leading to negative health consequences [5]. A recent study found that the severity of osteoarthritis could be promoted by air pollution via systemic inflammatory mechanisms [6]. In addition, pollution from traffic may be an environmental risk factor for rheumatoid arthritis [7]. An increased risk of rheumatoid arthritis in participants exposed to PM2.5 and NO_2_ was detected in a retrospective study in Taiwan [8]. Air pollution has been associated with the incidence of arthritis. However, whether this environmental factor is causally linked with osteoarthritis in humans, still remains a matter of debate. Muscular skeletal diseases such as knee osteoarthritis may develop over several decades and have been shown to be associated with biomechanical load during gait [9]. It has been shown that the knee adduction moment during gait leads to higher knee cartilage degeneration [10]. In addition, it has been shown that higher walking cadence is associated with less cartilage osteoarthritis progression [11]. Especially given that biomechanical data processing is challenging, the biomechanical research to date has tended to focus on small samples rather than multidisciplinary epidemiological research. Multidisciplinary epidemiological research can help control for psychosocial, physiological, or behavioral potentially biasing variables.

Active transport movements, air pollution, and health: A form of vigorous active transport is recreational running. Runners have a 30–45% lower risk of mortality and a reduced risk of cardiovascular, cancer, metabolic, mental, and neurodegenerative diseases [12]. However, not all people in the world have favorable conditions for physical activity such as running. In contrast with running health benefits, air pollutants have been proven to reduce life expectancy and induce respiratory, cardiovascular, cancer, lung and brain, diabetes, and dementia diseases [13]. A significant proportion of the active population is exposed to air pollution, particularly in large cities. Despite the increasing concentrations of air pollutants, nearly 30% of the male population and 20% of the female population participate in running as a form of exercise in the 18–29 age range. However, less than 2% of males and less than 0.7% of females continue to run past 65 years of age [14]. The causes of this decline in running participation, which is the most effective means of achieving longevity [12], remain unclear. Current investigation has shown that the doses of particulate matter exposure are a key factor for osteoarthritis severity under the jogging condition in rats [6]. In this context, the degradation of the musculoskeletal system may be one of the main reasons for the decline in participation in regular running in older age.

In addition, prospective cohort studies show that a minimum of 18% and a maximum of 92% of runners incur injuries during this activity [15]. The risk of injury and illness associated with running limits the use of the potential offered by this activity in health management for the current population. There are only a few prospective biomechanical studies focusing on running [15,16,17,18,19,20]. None of these studies apply control group, multidisciplinary approaches and the number of test subjects investigated is lower than 500. A prospective cohort study of a large number of runners, combined with a multidisciplinary approach (epidemiology, biomechanics, physiology, psychosociology), has the potential to reveal the causes of the most common chronic injuries in relation to air pollution. Among the most common injuries associated with running are Medial tibial stress syndrome, Achilles tendinopathy, Plantar fasciitis, Patellar tendinopathy, ankle sprains, and Iliotibial band syndrome [15]. All these injuries lead to a cessation of regular motion activity, and they may negatively influence adherence to physical activity.

During locomotion, it is often necessary to change direction or alter the direction of motion resulting from some external stimulus. However, such movements, often referred to as ‘cutting’, result in injuries particularly to the knee. A prominent knee injury, anterior cruciate ligament (ACL) rupture frequently occurs in non-contact, rapid movement situations such as cutting maneuvers in sport [21]. Females show greater ACL injury rates than males [22,23,24] and the prevalence of ACL injury increases over time in the female population [25]. Previous investigations [26,27] have shown that air pollution is associated with a decrease in neuro-behavioral functions and the level of gross and fine motor skills in children. Changes in neuro-behavioral function and the level of motor skills could influence associative risk factors of the ACL injury as well as movement coordination and its variability. Successfully negotiating a high ACL injury risk requires complex neuromuscular control strategies for dynamic joint stabilization [28]. Moreover, situational awareness, arousal, and attentional resources of the individual may influence areas of neurocognitive function, affecting the complex integration of vestibular, visual, and somatosensory information needed for neuromuscular control [28].

Aims of the Study: The general aim of the 4HAIE study is to investigate the influence of air pollution on the incidence of sports related injuries, physical activity related injuries, physical (in)activity, health, and quality of life across the lifespan. The specific purpose of the biomechanical and musculoskeletal protocol of the 4HAIE study is to investigate the potential impact of a polluted environment on the biomechanics of human active transport including the quality of knee cartilage, Achilles tendon and ACL determined by magnetic resonance imaging (MRI). We hypothesize that there will be interactions of environmental, biomechanical, physiological, and psychosocial variables and that these interactions will cause musculoskeletal diseases/protection.

## 2. Material and Methods

### 2.1. Design

**Figure 1:**
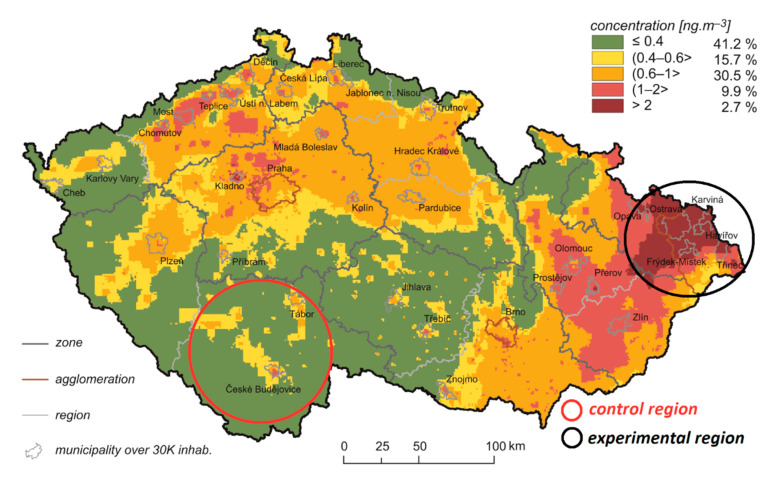
Field of annual average concentration of benzo[a]pyrene for 2018. The figure is modified according to Air Quality Information System—Czech Hydrometeorogical Institute [33].

A total of 1500 active runners and inactive controls aged 18–65 will be recruited for this study. Inactive controls are defined as those who can run but choose not to run and do not meet public health recommendations for physical activity. In total, 900 physically active runners and 600 inactive controls, both men and women, will be recruited, with equal representation of those living in the experimental highly-polluted industrial region (*n* = 750) and the control region with low pollution levels (*n* = 750) (Figure 1). The available data on air pollution in Czechoslovakia, and later the Czech Republic, have been collected since the beginning of the 1950s. Due to long-term monitoring results of ambient benzo[a]pyrene and particulate matter pollutants, the Moravian-Silesian region of the Czech republic has been recognized as a European hotspot [29]. Exposure to benzo[a]pyrene in Ostrava–Radvanice has been the highest in the European union for a decades [30]. Muscular skeletal diseases such as osteoarthritis may develop over several decades [9]. We therefore chose the highly and long-term polluted region of Moravia-Silesia as the experimental region. Due to relatively low concentrations of pollutants in the air in the region of South Bohemia from 1950 to the present, this region was chosen as the control region [29,30,31]. The South Bohemian region has been shown to be significantly less polluted than Moravian-Silesian region from the long-term perspective [29,30]. Both regions are comparable with respect to ethnicity with the majority of population (ca. 95%) being White Caucasian and ascribing to either Czech or Moravian nationality (both speaking the Czech language and being of similar cultural and ethnical background) [32].

Participants will be recruited through a professional social science and marketing research company. The sample will be recruited using quota sampling based on location, age, gender, and activity status (reflecting the sociodemographic distribution of the specific populations). Interested participants will be screened online and by telephone. Eligible participants complete online questionnaires and undergo a 2-day baseline laboratory assessment, including biomechanical, physiological, psychological testing, and imaging. Subsequently, participants will be monitored for one year after baseline testing. During this year, objective physical activity data will be collected through Fitbit Charge 3 monitors, along with data on incidence of injuries, air pollution, psychological factors, and behavior collected through surveys on a custom designed mobile application. Data collection began in April 2019 and is expected to be completed by April, 2021. Ethical approval was obtained from the Ethics and Research Committee of the principal author university and all participants are required to sign an informed consent form prior to data collection.

#### 2.1.1. Inclusion Criteria for Active Runners

Active runners must spend at least 150 min a week in moderate-intensity physical activity or 75 min in high-intensity physical activity (or an equivalent combination of moderate- and vigorous-intensity activity), including running [34]. They must run regularly for 6 weeks or longer, at least 10 km per week, and plan to continue running for another 12 months. Participants must also be between 18–65 years of age upon enrollment, reside in the given localities year-round for at least 5 years, and have no plan to move from the given locations in the next 12 months. Participants must have internet access and own a smartphone (with Android 5.0 or higher, or iOS operating systems) with a data connection (Wi-Fi or mobile data).

#### 2.1.2. Inclusion Criteria for Inactive Controls

Inactive controls fulfill the same criteria as active runners, but, in spite of being otherwise capable of physical activity including running (i.e., having no limitations to physical activity diagnosed by their physician), they do not meet public health recommendations for physical activity (i.e., engage in physical activity for less than 150 min a week).

#### 2.1.3. Exclusion Criteria

Participants are excluded if they are smokers and/or report experiencing acute problems that hinder physical activity in the last six weeks (surgery, pain, injury) or experience any other acute illness. Those with other chronic disease conditions may participate only after presenting a written consent from their physician. Physician’s consent is also required if indicated by the Physical Activity Readiness Questionnaire (PAR-Q) screening test. Additional exclusion criteria involve contraindications to magnetic resonance imaging or dual-emission X-ray absorptiometry (DXA) examination (e.g., pregnancy, radiological examination in the last 7 days using iodine/barium contrast agents, pacemaker, radioactive body, surgical staples, insulin pump, cochlear implant, other metal implants, and foreign bodies such as shrapnel, etc.).

### 2.2. Experimental Set-Up

#### 2.2.1. Biomechanical Set-Up

Overground walking, running, cutting and treadmill running kinematics of the lower extremities are recorded using a high-speed motion capture system. Treadmill running is acquired using a 9-camera motion capture system (8x Oqus 100 and 1x Oqus 510+, Qualisys, Inc., Gothenburg, Sweden). Overground walking, running and cutting is acquired using a 10-camera motion capture system (9x Oqus 700+ and 1x Oqus 510+, Qualisys, Inc., Gothenburg, Sweden). Three force plates (Kistler 9286AA, 9281CA and 9287CCAQ02, Kistler Instruments AG, Winterthur, Switzerland) are used to collect ground reaction force (GRF) data. The force plates are built into a 17 m long runway. In addition, treadmill running GRF is recorded using a treadmill with an embedded force platform (Bertec, USA). Kinematics and ground reaction force data is sampled at a frequency of 240 Hz and 1200 Hz (treadmill) and 2160 Hz (force plates) respectively. Overground running speed is controlled using two photocells (OPZZ, EGMedical s.r.o., Brno, Czech Republic) located at intervals of 3 m along the runway. Cutting maneuver maximal approach speed is controlled using two photocells (P-2RB/1, EGMedical, Ltd., Czech Republic) located at intervals of 2 m along the runway. Electromyographic (EMG) data is collected using a Delsys Trigno Wireless EMG System sampling at 20–450 Hz (Delsys Inc., Boston, MA, USA).

#### 2.2.2. Magnetic Resonance Imaging Set-Up

Magnetic resonance data will be acquired at a 1.5 T Siemens Magnetom Sempra Scanner (Siemens, Erlanger, Germany). A 12-channel transmit/receive knee coil is used to obtain images of the knee. The knee protocol includes six sequences: Proton density (PD)-weighted turbo spin echo (TSE) fat-suppressed (FS) in transversal (TRA), coronal (COR) and sagittal (SAG) plane, COR T1-weighted TSE, 3D Dual-echo Steady-State (DESS) water excitation (WE), SAG T2 mapping for knee cartilage, para-sagittal T2 mapping for ACL and 3D flow phase contrast (PC) for MR angiography (MRA) of the knee vessels. To obtain the highest quality images of the Achilles tendon (AT), a 16-channel transmit/receive head coil is used. The ankle protocol includes five sequences: SAG T1-weighted spin echo (SE), TRA T2-weighted TSE, COR T1-weighted TSE, SAG and TRA PD-weighted TSE FS, and SAG T2* mapping. The imaging parameters of the sequences are described in subsection Detailed magnetic resonance imaging protocol.

### 2.3. Protocol

#### 2.3.1. Global 4HAIE Protocol and Interactions

Prior to the laboratory visit, each participant will complete two sets of baseline socioeconomic and psychological questionnaires using the online Qualtrics platform (Qualtrics, Provo, UT, USA). The participants from South Bohemian control region will be transported 384 km to the Human Motion Diagnostic Center located in the experimental air-polluted (Moravian-Silesian) region by a laboratory car. Informed consent will be administered in person prior to the start of laboratory assessments.

On the first day of the laboratory visit, each participant is familiarized with the questionnaire smartphone application and the Fitbit device. Following this, participants complete a physical activity questionnaire, and a hair sample is collected. Blood pressure measurements, a graded exercise test, and respiratory capacity test are then performed. After dinner, each participant follows a standardized protocol for sleep assessment collected at the sleep laboratory.

The second day of laboratory visit starts with heart rate variability measurement, a questionnaire assessing the quality of sleep and an injury and pain survey. Prior to the physiological testing, each participant will complete fatigue questionnaires. During physiological testing, blood sample intake, bioimpedance measurements, and somatic measurements will be collected. After breakfast, cognitive function testing, DXA, and magnetic resonance imaging of the right ankle, right knee, and brain is performed. Finally, functional lower extremity tests and biomechanical testing are performed by each participant.

These baseline measurements are followed up for one-year using an online evaluation of physical activity through the Fitbit device, behavioral measures through a smartphone questionnaire app, as well as passively evaluated air pollution exposure. In addition, ecological momentary assessment is used and the pain and history of any running-related injury are evaluated through a brief survey administered on their smartphones.

#### 2.3.2. Biomechanical Protocol

**Figure 2:**
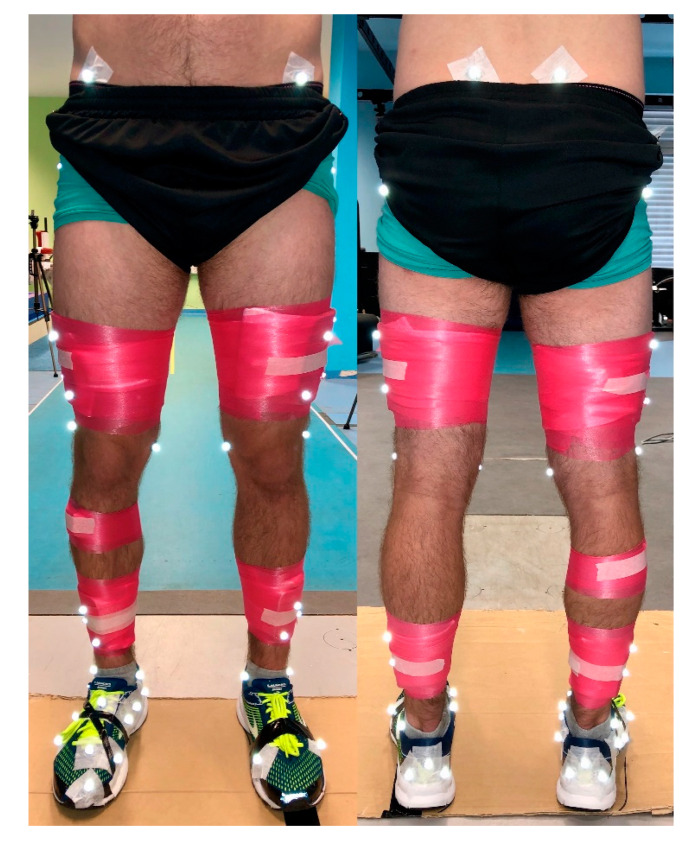
Reflective calibration and tracking markers placement and EMG sensors placement for biomechanical analysis.

Overground walking, running and treadmill running trials are completed in a counter-balanced order dependent on the number of participants during particular testing day. Subsequently, a cutting maneuver are performed. Reflective calibration and tracking markers are positioned on the pelvis bilaterally on the posterior superior iliac spines, and the anterior superior iliac spines. Markers are also positioned bilaterally on the lower limb on the medial and lateral malleoli, the medial and lateral femoral condyles, the greater trochanter of the femur and on four light-weight rigid plates with four markers per plate on the thigh and shank [35]. Markers are used to define the multi-segmental Rizzoli foot model on the right foot [36,37]. On the left foot rigid body model, a triad on the heel and markers on the first and fifth metatarsal heads are placed. Markers are attached on standard laboratory neutral running shoes (Brooks Launch 5, Brooks Sport Inc., Seattle, WA, USA). Delsys EMG sensors are placed over skin on the following muscles: rectus femoris, biceps femoris, gluteus medialis, and tibialis anterior (Figure 2). Prior to the biomechanics measurements, a standing calibration trial is recorded followed by a half-squat for EMG calibration.

Before measuring the biomechanics of running, the self-preferred speed is set. For runners, the question is: What is your usual speed when you go jogging for 45 min? Non-runners are asked to set the running speed at a pace that will allow them to run as far as possible (if they are unable to imagine the running pace for 45 min). Then, the participant runs at this pace for 2 min and in the last 30 s we measure the speed of 4 runs with photocells. The average of the four runs then indicates their self-preferred speed. Overground running consists of eight successful trials on a 17 m long runway at the participant’s self-preferred speed ±5%. Six successful trials at self-preferred speed will be collected for overground walking. The starting position for running trials is always set up at least (e.g., 7 m) from the force plate and for walking (e.g., 5 m). A successful walking/running trial consists of a participant landing on the force plate with the whole right foot. The subject’s starting position is fine-tuned so that they land in the middle of the pressure plate while walking normally at preferred velocity [38]. Additionally, subjects are instructed to focus on a distant point at eye level while walking, not on the pressure plate [39]. Participants run on the treadmill for 2 min at a fixed speed of 2.75 m/s. If the velocity corresponding to participant’s second ventilatory threshold (VT2) value from GXT is lower than 2.75 m/s, their speed is adjusted as follows: VT2 velocity is multiplied by 0.8. Subsequently, the speed of the belt is increased to 3.0 m/s or to the speed corresponding with the second ventilatory threshold from the VO_2_max test. The order of these two conditions is chosen depending on the speed (ascending). Both speed intervals are one-minute long with 30 s of recording.

**Figure 3:**
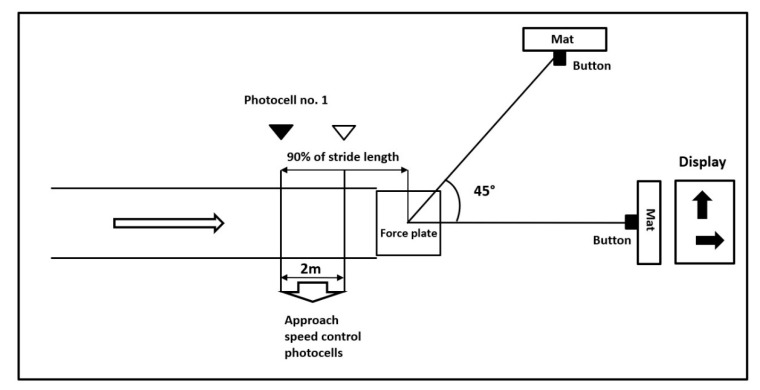
Unanticipated cutting maneuver scheme.

Lastly, an unanticipated cutting maneuver is evaluated. For easy identification of the direction, a reflective tape is attached to the floor at an angle of 45° to the left side. A maximal approach speed is controlled using two photocells. For the unanticipated sidestepping task, photocell number 1 triggers a visual display to indicate if the participant will conduct a sidestep cut or proceed straight ahead. Photocell number 1 is placed at a calculated distance from the center of the force plate (90% of stride length plus an added distance for correction for the timekeeping signal delay based on the approach speed—0.2 s) (Figure 3). Participants are required to perform five successful running trials and five cutting unanticipated trials at the maximal self-preferred speed and then touch the mat at the end of the track as quickly as possible. Five successful cutting trials are collected after unanticipated trials presented in random order.

#### 2.3.3. Magnetic Resonance Imaging Protocol

In our protocol, each participant recruited for the study undergoes a right knee and right ankle scan. Firstly, the participant is asked to sign an informed consent form and is fully informed about the procedure of the examination. Subsequently, the participant is positioned feet first in supine position on the bed of the MRI scanner. The right knee joint is placed in the 12-channel knee coil and iso-centered in the scanner. The knee joint and ACL is evaluated qualitatively and quantitatively, and the total acquisition time (TA) is approximately 25 min.

In order to quantitatively analyze the knee cartilage and anterior cruciate ligament (ACL), the T_2_ mapping sequence in the SAG plane is used. The sequences for qualitative evaluation of the knee includes PD-weighted sequences in all three planes, T1-weighted in COR plane, 3D T2-weighted and 3D flow MRA. Imaging parameters for MRI sequences used for the knee are described in Table 1. The ankle joint scan with the main focus on the AT is performed in a 16-channel head coil with the designed device for fixing the leg at a 90-degree angle. Participants lie in a supine position with their ankle fixed and are asked to remain completely still during the scan. The acquisition time is approximately 20 min (Table 2).

#### 2.3.4. Functional Testing Protocol

Each participant will complete the following 5 physical examinations used in clinical practice: (i) Trendelenburg test for identification of hip abduction weakness during a single-leg stance position for 30 s. The pelvic-on-femoral position of the stance lower extremity is measured as the angle (°) between the anterior superior iliac spine and longitudinal axis of the femur for both side [40,41]. (ii) Knee anterior drawer test is used to test the laxity of the knee’s anterior cruciate ligament (ACL). The participant lie supine, hip flexed to 45°, knee flexed to 90°. Anterior—posterior displacement of the tibia relative to the thigh will be measured [42]. The displacement is graded relative to the contralateral side. The displacement is evaluated based on International Knee Documentation Committee (IKDC) categories as normal (-), nearly normal (+), abnormal (++), and severely abnormal (+++) [43]. (iii) Ankle anterior drawer test is used to test the ligament of the anterior talofibular. The participant lies supine, ankle in 10–15° plantar flexion. The posterior displacement of the heel relative to the distal tibia is measured [44]. The displacement is graded relative to the contralateral side. The participants are classified into three grades. Grade I (-) is defined as a stable joint, grade II is a partially unstable joint with two different displacements of the heel relative to the distal tibia (+); (++) and grade III is a completely unstable joint (+++). (iv) The lunge test is used to assess the dorsiflexion range of the movement. The participant is in a standing position facing a wall. Tested foot and knee are perpendicular to a wall, the opposite limb is in tandem position. Participants are instructed to perform a lunge when the knee is in contact with the wall while the heel is still in contact with the ground. The distance between the great toe and the wall is measured (cm) [45]. (v) The kick a ball test determines functional dominance. The participant kicks a ball 3 times into the marked goal area. The lower extremity that is used for most trials is identified as the dominant limb [46].

### 2.4. Data Analysis

#### 2.4.1. Biomechanics Data Analysis

The kinematic and kinetic data is processed using QTM (Track Manager; Qualisys, Sweden, Göteborg) and Visual3D software (C-Motion, Germantown, Kentucky, KY, USA,). Walking, running and cutting main gait events are based on the automatic gait event identification [47]. Ground-reaction force and marker kinematic data is filtered using a fourth-order Butterworth low-pass filter with a cut-off frequency of 50 Hz (analog ground reaction force data) and 12 Hz (kinematics) for overground running trials, 40 Hz and 8 Hz for treadmill running and overground walking trials, and 50 Hz and 15 Hz for cutting trials [48]. This cut-off frequency was selected based on previous literature, residual analysis and visual inspection of pilot data [48,49,50,51]. The proximal and distal ends and the coordinate systems of the lower extremity segments and pelvis are determined from the calibration trial [52]. Hip, knee, and ankle three-dimensional joint angles are calculated using an x-y-z Cardan rotation sequence. Angles in the lower extremity joints are determined throughout the entire stance phase and whole gait cycle. The three-dimensional net internal ankle, knee, and hip joint moments are calculated using a Newton–Euler inverse dynamics technique [53].

Electromyography (EMG) patterns are recorded during the overground walking and running and cutting trials using surface electrodes (Delsys Inc., Boston, MA, USA). Data from major surface muscles of the lower extremities, rectus femoris, biceps femoris, gluteus medialis, and tibialis anterior are collected. The raw EMG signals from all type of movement are processed using a band-pass filter (50–450 Hz), rectified, and consequently passed through a critically damped low-pass filter with a 20 Hz cut-off [54]. The signal is then normalized to the peak processed signal found during standardized squat.

#### 2.4.2. Magnetic Resonance Imaging Data Analysis

Semi-quantitative assessment of cartilage on MRI images is performed by an expert musculoskeletal radiologist (M.G., 10 years of experience) using the modified Outerbridge grading of chondromalacia [55]. This grading system is divided into five grades by MRI (0—physiological state, 1—focal areas with hyperintensity, 2—swelling of articular cartilage extending to surface, 3—partial-thickness cartilage loss, and 4—full-thickness cartilage loss). The modified Outerbridge grading of chondromalacia is widely used for morphological analysis of cartilage in clinical trials as well as in epidemiological studies [56,57,58]. The cartilage assessment is conducted in 14 anatomical regions (two patellar, six femoral, and six tibial): lateral/medial patella, lateral/medial anterior femur (AF), lateral/medial central femur (CF), lateral/medial posterior femur (PF), lateral/medial anterior tibia (AT), lateral/medial central tibia (CT), lateral/medial posterior tibia (PT). The ACL is assessed semi-quantitatively as follows: 0—physiological state, 1—primary signs of ACL contusion (swelling, fiber discontinuity), 2—partial ACL tear, and 3—total ACL tear [59,60]. To evaluate the AT, all subjects are rated using Vienna morphological achilles tendon score (VIMATS; 0–100, worst to best) [61]. As reported by Apprich et al. [61], thickness, continuity, signal intensity, and associated pathologies is evaluated.

For the quantitative analysis, T2 maps of knee cartilage and ACL are calculated by mono-exponential fitting of the signal intensity decays on a pixel-by-pixel basis. Knee cartilage and ACL is manually segmented from T2-weighed images by two readers blinded to morphological findings (K.B., D.V. supervised by V.C.) using a segmentation and analysis tool built using MATLAB software (Mathworks Inc., Natick, MA, USA). Knee cartilage is divided into regions-of-interest (ROIs) similar to the regions used in the modified Outerbridge grading of chondromalacia. A similar set up of quantitative analysis as reported in [62,63] is used. ACL is divided into four regions-of-interest (ROIs): insertion, middle, upper, and whole ACL. Average T2 relaxation times of cartilage and ACL are calculated for each ROI.

In order to quantitatively assess the AT, ROIs are manually drawn from sagittal images by two readers (V.H. and D.V., supervised by V.J.) in three regions: insertion part (INS), middle part (MID), and muscle-tendon junction (MTJ). The ROIs are drawn in MatLab (MathWorks, Natick, MA, USA). Total length of each part is selected as one-third of the total length of AT. Values from ROIs are analyzed by mono- and bi-exponential fitting procedure in Interactive Data Language (IDL 6.3, Boulder, CO, USA). Set-up of these analyses is described in more detail in [64].

## 3. Recruitment and Data Example

**Figure 4:**
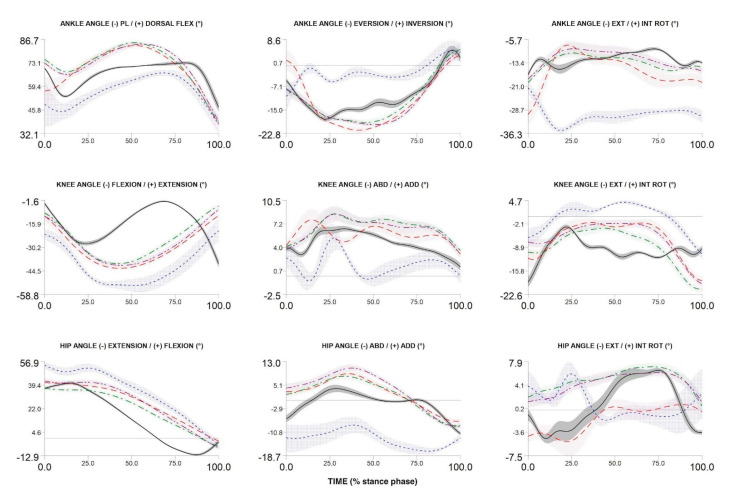
Three-dimensional ankle, knee and hip angles during stance phase of walking, over-ground running, treadmill running (3.0 m/s and 75% of maximum VO_2_max test speed) and cutting maneuver in one participant. Over-ground running—red dash line; walking—black solid line; cutting maneuver—blue dot line; TR3—green dash dot line; TRA—purple dash dot dot line.

**Figure 5:**
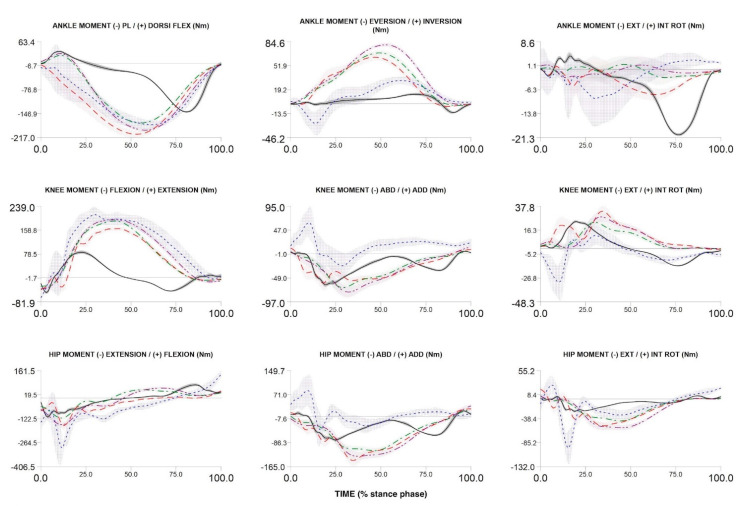
Three-dimensional ankle, knee and hip net moments during stance phase of walking, over-ground running, treadmill running (3.0 m/s and 75% of maximum VO_2_max test speed) and cutting maneuver in one participant. Over-ground running—red dash line; walking—black solid line; cutting maneuver—blue dot line; TR3—green dash dot line; TRA—purple dash dot dot line.

**Figure 6:**
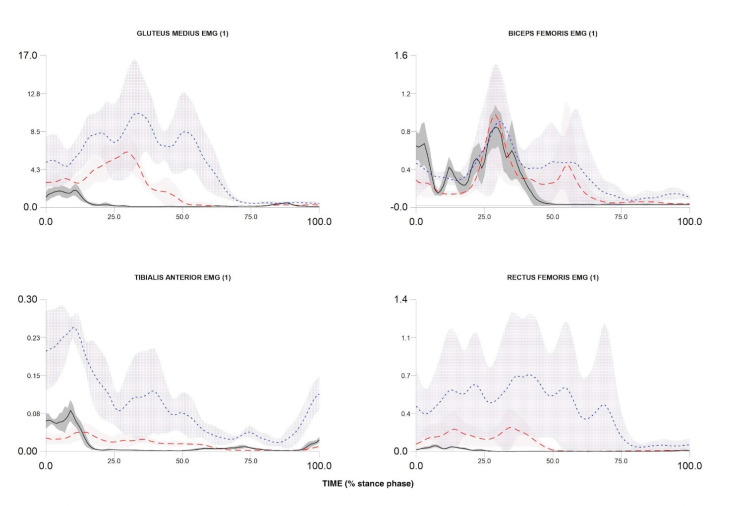
Electromyography activity during stance phase of walking, over-ground running and cutting maneuver in one participant. Over-ground running—red dash line; walking—black solid line; cutting maneuver—blue dot line.

To date, we have recruited and measured 1024 participants. Figure 4, Figure 5 and Figure 6 compare the kinematic, kinetics and electromyography results obtained from the preliminary biomechanical analysis of walking, running, and cutting movements for sample participant. The average angles, moments, and EMG curves make it possible to clearly distinguish walking from running and cutting maneuvers trials (Figure 4, Figure 5 and Figure 6).

**Figure 7:**
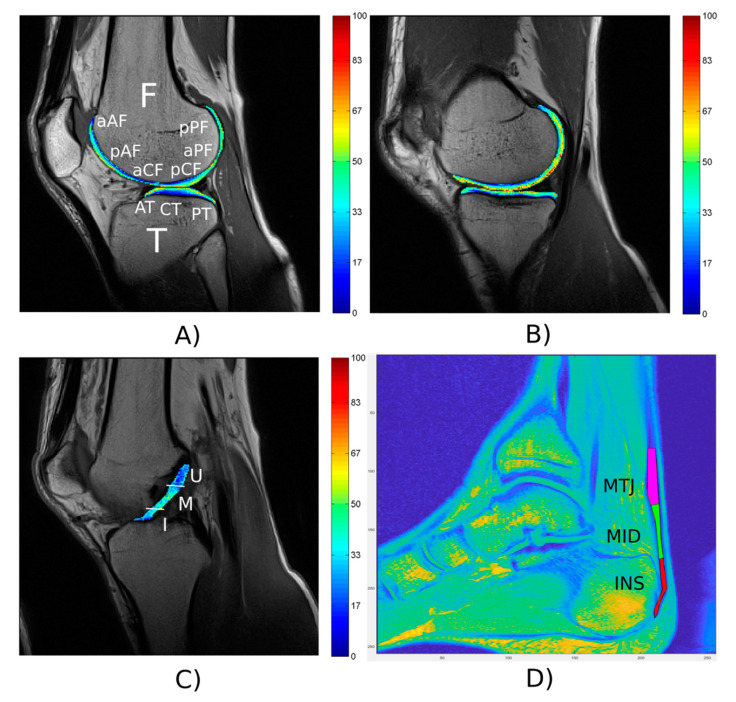
Representative T2 relaxation time map of knee articular cartilage and regions-of-interest (ROIs) for the lateral (**A**) and medial compartment (**B**); F—femur, aAF—anterior side of anterior part of femur, pAF—posterior side of anterior part of femur, aCF—anterior side of central part of femur, pCF—posterior side of central part of femur, aPF—anterior side of posterior part of femur, pPF—posterior side of posterior part of femur, T—tibia, AT—anterior part of tibia, CT—central part of tibia, PT—posterior part of tibia. Representative T2 relaxation time map and ROIs of ACL (**C**); Whole ACL, I—insertion, M—mid part, U—upper part). ROIs of AT on sagittal T2*-weighted image (**D**); INS—insertion, MID—middle, MTJ—muscle-tendon junction.

The results obtained from the MRI analysis of one ankle and one knee are shown in Figure 7. The results indicated that the T2 relaxation times of the knee cartilage were within normal physiological ranges (Figure 7A,B). The qualitative evaluation of knee cartilage according to the modified Outerbridge grading of chondromalacia was determined by the radiologist for all evaluated regions as physiological state (grade 0). Only the region of patellar cartilage showed focal areas with hyperintensity (grade 1). The ACL was qualitatively assessed by a radiologist as no degeneration (grade 0), which is consistent with the quantitative analysis of ACL in Figure 7C. T2* mono-exponential relaxation times for AT were determined for bulk 8.25 ± 6.67 ms, insertion 7.05 ± 4.59 ms, midportion 10.28 ± 3.21 ms and musculoskeletal junction 7.74 ± 3.33 ms. The VIMATS AT score was determined by radiologist (total 95 points from 100: thickness 20 from 20, continuity 30 from 30, signal intensity 20 from 20, associated pathologies 25 from 30) and corresponded to the Achilles tendon without degeneration (Figure 7D).

The results of the one participant are presented for each clinical test: (i) Trendelenburg test, right side (0°), left side (0°); (ii) Knee anterior drawer test, right knee (-), left knee (-); (iii) Ankle anterior drawer test, right ankle (-), left ankle (-); (iv) Lunge Test, right lower limb (12 cm), left lower limb (12 cm); (v) Kick a ball test, dominant limb (right).

## 4. Discussion

We introduced the biomechanical and musculoskeletal imaging protocol designed to investigate the musculoskeletal and neuro-mechanical health in the 4HAIE human cohort. It has been suggested [65] that the last 45 years of research on running musculoskeletal injuries tended to focus only on mechanical loading while the musculoskeletal tissues properties and the specific social and environmental factors have been neglected [65]. The protocol for this study might reveal new knowledge, especially in the following globally investigated issues related to the health and quality of life of the population.

Ecological momentary assessment and one-year online monitoring using IT technologies will allow us to uniquely examine the interplay of biomechanical, physiological, anatomical, psychosocial, and environmental factors affecting running injuries. Although research on running injuries may seem like a negligible reason for such an extensive study, it is muscle skeletal injuries that can be the cause of a significant decline in physical activity during older adulthood [12]. Secondly, we would like to investigate the possible associations between physical activity, air pollution and musculoskeletal tissues degeneration (knee cartilage, ACL, and AT). Only such an extensive study, which also examines psychosocial and physiological covariates, can indicate whether the conclusions about the impact of air pollution on musculoskeletal health in previous animal studies and uncontrolled human studies are robust [3,6].

One of the possible examples of hypotheses which could be evaluated based on this one-year prospective study is that the external environment (air pollution) will influence the incidence of running related injuries. Interactions between psychosocial and physiological variables may exist, and these interactions may result in a higher incidence of running injuries especially in the air polluted region compared with the control region. Another possible hypothesis which could be addressed based on baseline cross sectional study is that the decrease in physical activity in older age may be related to gait biomechanics and the degeneration of dominant musculoskeletal structures such as knee cartilage and Achilles tendon. Moreover, we hypothesize that knee cartilage and Achilles tendon degeneration related to aging may be associated with a healthy lifestyle and environment. Specifically, we hypothesize that runners in the air polluted region will have different properties of knee cartilage and Achilles tendon compared with inactive controls and this difference between runners and inactive individuals will not be equally large in the control unaffected region. It can also be determined whether AT and knee cartilage degeneration is associated with running/in-activity status and whether air pollution status affects this association. We can adjust for potential confounders in statistical analyses: biomechanical load, physical activity, aerobic fitness, history of running related injury, etc.

The patterns of kinematics, kinetics and electromyographic activity during walking, running and cutting maneuvers allow a comprehensive description of possible biomechanical loads during locomotion and change of direction. The measured curves are in accordance with the results presented in previous studies [54,66,67,68,69]. In addition, the ranges and maxima of angles, moments, and EMG are in accordance with the intensity of individual movement tasks (walking lowest intensity, running medium intensity, and cutting maximum intensity). Discrete biomechanical variables derived from the presented curves (e.g., initial contact angles, maximum moment values, net joint powers, coordination variability, etc.) can be used in multidimensional statistical models to identify factors affecting musculoskeletal injuries.

We verified the health of the sample participant as evident from the cartilage, ACL and AT to be in physiological state without pathologies. Values of T2 relaxation time of cartilage in the presented participant agreed with modified Outerbridge grading of chondromalacia. Even relaxation times for ACL and qualitative assessment by radiologist did not indicate degeneration. T2 relaxation time has been shown to be sensitive to cartilage biochemical and biomechanical properties [70], and has been associated with morphological abnormalities of cartilage and bone [63,71]. Knee cartilage transverse relaxation time (T2) mapping is sensitive to cartilage damage and quality of cartilage collagen and hydration [72]. Quantitative ACL T2 relaxation time has potential for non-invasive identification of ACL degeneration [73]. A shorter T2* component correlated strongly with clinical score of the Achilles tendon degeneration [64]. Thus, these methods can help us to objectively quantify the quality of cartilage, ACL, and AT tissue. Quantitative MRI methods can play a key role in research into the development of musculoskeletal injuries.

The research to date has tended to focus only on biomechanical load rather than the multidisciplinary approach to the identification of the causes of running related injuries and severe musculoskeletal acute and chronic problems [65]. A strength of this study is also the prospective one-year monitoring of running related injuries and problems using modern IT technologies. The uniqueness of this study lies in the large cohort of active and inactive individuals from air-polluted and control region with low pollution levels. However, the project is limited by its cross-sectional design of research with regard to serious musculoskeletal disorders, such as osteoarthritis. Further longitudinal follow-up measurements will be conducted after five years if an association between the biomechanical load and osteoarthritis or the external environment is demonstrated. A prospective design would then help to understand the causes of musculoskeletal diseases, as well as the importance of these diseases in terms of decreased physical activity with age.

## 5. Conclusions

Herein, we introduced a biomechanical and musculoskeletal protocol with the example data of one healthy participant to investigate the musculoskeletal and neuro-mechanical health in the 4HAIE cohort, including the design for controlling for physiological and psychological injury factors. In the current ongoing research project, we hypothesize that there will be interactions of biomechanical, physiological, and psychosocial variables and that these interactions will cause musculoskeletal diseases/protection, especially in the highly polluted industrial region compared to the unpolluted control region.

## Figures and Tables

**Table 1:** Imaging parameters for magnetic resonance imaging sequences used for morphological evaluation of the knee.

Imaging Parameter	Axial PD TSE FS	Coronal PD TSE FS	Sagittal PD TSE FS	Coronal T1 TSE	3D DESS WE	Sagittal T2 Map Cartilage	Sagittal T2 Map ACL	3D PC MRA
Repetition time (ms)	4510	5760	4250	490	18	1690	1500	117
Echo time (ms)	27	25	26	12	7.05	12, 24, 36, 48, 60	12, 24, 36, 48, 60	18.7
Slice thickness (mm)	3	3	3	3.2	0.6	3	3	1.1
Field of view (mm)	150	160	160	160	150	160	160	220
Matrix size	198 × 256	235 × 320	212 × 320	223 × 320	230 × 256	256 × 256	256 × 256	144 × 256
Flip angle (°)	150	150	150	150	25	180	180	15
Inter-section gap (mm)	0.3	0.3	0.3	0.6	0.1	0.6	0.6	0.2
Bandwidth (kHz)	140	145	140	150	260	230	225	320
Echo train length	8	10	8	2	2	5	5	0
Signal average	1	1	1	1	1	1	1	1
Number of slices	30	35	30	28	160	18	5	80
Acquisition time (min:s)	2:08	2:08	2:09	1:36	3:41	3:55	3:29	6:33

**Note:** PD, proton density; TSE, turbo spin-echo; FS, fat saturation; DESS, Dual-echo Steady-State; PC, phase contrast.

**Table 2:** Imaging parameters for magnetic resonance imaging sequences used for morphological evaluation of the ankle.

Imaging Parameter	Axial T2 TSE	Sagittal T1 SE	Coronal T1 TSE	Sagittal PD TSE FS	Axial PD TSE FS	Sagittal T2* Map Short	Sagittal T2* Map Long
Repetition time (ms)	4000	555	610	4620	5200	485	485
Echo time (ms)	61	22	11	39	36	3.78, 0.77, 17.15, 23.52, 29.89	7.28, 14.28, 21.28, 28.28, 35.28
Slice thickness (mm)	3	3	3	3	3	3	3
Field of view (mm)	160	410	160	160	135	160	160
Matrix size	156 × 196	448 × 448	180 × 320	240 × 320	230 × 256	205 × 256	205 × 256
Flip angle (°)	150	90	150	150	150	60	60
Inter-section gap (mm)	0.6	0.6	0.6	0.6	1	0.6	0.6
Bandwidth (kHz)	210	190	190	200	220	415	415
Echo train length	12	1	3	12	10	5	5
Signal average	1	1	1	1	1	2	2
Number of slices	26	18	28	25	35	11	11
Acquisition time (min:s)	0:58	6:27	1:23	1:25	2:17	3:20	3:20
